# Adult-onset, short-term dietary restriction reduces cell senescence in mice

**DOI:** 10.18632/aging.100196

**Published:** 2010-09-11

**Authors:** Chunfang Wang, Mandy Maddick, Satomi Miwa, Diana Jurk, Rafal Czapiewski, Gabriele Saretzki, Sabine A.S. Langie, Roger W.L. Godschalk, Kerry Cameron, Thomas von Zglinicki

**Affiliations:** ^1^ Centre for Integrated Systems Biology of Ageing and Nutrition, Institute for Ageing and Health, Newcastle University, Newcastle Upon Tyne, UK; ^2^ Crucible Laboratory, Institute for Ageing and Health, Newcastle University, Newcastle Upon Tyne, UK; ^3^ Human Nutrition Research Centre and Centre for Brain Ageing and Vitality, Institute for Ageing and Health, Newcastle University, Newcastle Upon Tyne, UK; ^4^ Nutrition and Toxicology Research Institute Maastricht (NUTRIM), Department of Health Risk Analysis and Toxicology, Maastricht University, Netherlands

**Keywords:** Dietary restriction, caloric restriction, mice, senescence, telomeres, ageing

## Abstract

Dietary restriction (DR) extends the lifespan of a wide variety of species and reduces the incidence of major age-related diseases. Cell senescence has been proposed as one causal mechanism for tissue and organism ageing. We show for the first time that adult-onset, short-term DR reduced frequencies of senescent cells in the small intestinal epithelium and liver of mice, which are tissues known to accumulate increased numbers of senescent cells with advancing age. This reduction was associated with improved telomere maintenance without increased telomerase activity. We also found a decrease in cumulative oxidative stress markers in the same compartments despite absence of significant changes in steady-state oxidative stress markers at the whole tissue level. The data suggest the possibility that reduction of cell senescence may be a primary consequence of DR which in turn may explain known effects of DR such as improved mitochondrial function and reduced production of reactive oxygen species.

## INTRODUCTION

Dietary restriction (DR), whereby total caloric intake is reduced but adequate nutrition is maintained, results in an extension of lifespan. Additionally, DR has been shown to delay the onset and severity of cancer and other diseases associated with ageing [1]. The DR response has been remarkably robust in a wide range of animal species, although both evolutionary models and genetic experiments question its universality in different inbred strains of mice and, importantly, in humans [2,3]. The molecular and cellular mechanisms underlying the response to DR have been intensely examined. It has been proposed that DR prolonged lifespan for example by attenuating oxidative damage, reducing production of reactive oxygen species (ROS), increasing DNA repair capacity, altering the growth hormone/IGF-1 axis, decreasing signaling through the mTOR substrate S6K1 or improving hormesis [4-8]. However, we are still far from a mechanistic and integrative understanding of the DR response [1,9].

This is even more true for the response to adult-onset, short-term DR. While the effect on lifespan becomes less robust if DR is implemented in older animals [10,11], there are still strong beneficial effects on cancer incidence [11-15], immune response [14,16] and cognitive function [17]. Preliminary data from non-human primates [18] and clinical trials [19] have suggested that late onset DR could have at least some beneficial effects in humans.

Recently, evidence is mounting that cellular senescence, which was originally described as the permanent loss of replicative capacity in human fibroblasts *in vitro* [20], is a complex phenotype, possibly causally contributing to aging *in vivo* [21-23]. Senescent cells are found with increasing frequency in many tissues of aging rodents, primates and humans [24-28].High frequencies of senescent cells have been associated with age-related diseases like osteoarthritis and atherosclerosis [29,30] and were also found in mouse models of accelerated aging [31-34]. Senescent cells are not simply incompetent of proliferating; they display major alterations to their gene expression profiles [35] and secrete bioactive molecules including matrix-degrading enzymes [36], inflammatory cytokines [21,22,37] and ROS [23]. Thus, cell senescence may well be an important driver for the aging process *in vivo* [38,39].

If this concept were correct, one would hypothesize that a reduction of cell senescence might be part, and potentially a causal part, of the beneficial action of DR. This would be interesting because less senescent cells could explain the anti-inflammatory and anti-oxidative action of DR. However, there are few data to support such a hypothesis. There is good evidence that both life-long and adult-onset DR limit T cell senescence in mice and primates [14,40-42], at least partially by maintaining sensitivity to stress-induced apoptosis [43]. However, T cell senescence might be very different from senescence of cells in solid tissues. For instance, while a DNA damage response is the major driver for growth arrest [44] and phenotypic changes [23] in fibroblast senescence, its role in T cell senescence is less well established. Moreover, sensitivity to apoptotic stimuli is generally high in senescent T cells, but decreases during senescence in fibroblasts and other solid tissue cells.

There is very little data available on the impact of DR on cell senescence in solid mammalian tissues. Early data [45,46] showed reduced proliferative activity in various tissues of young mice under DR but improved maintenance of replicative activity and capacity in old mice under life-long DR, which might be due to a decreased accumulation of senescent cells. Krishnamurthy et al. [26] showed that DR reduced staining for senescence-associated β-Galactosidase (sen-β-Gal) and the expression of p16^INK4a^ and p19^Arf^ in the kidney. However, the specificity and sensitivity of sen-β-Gal as a marker for senescent cells *in vivo* has been repeatedly questioned [47,48]. Moreover, p16^INK4a^ and p19^Arf^ expression was similarly changed in postmitotic tissues like brain cortex and heart, suggesting that expression from the INK4A locus might be a better indicator for aging than for cell senescence. Further indirect evidence for decreased cell senescence under DR came from a study showing reduced levels of IGFBP3, a major secretion product of senescent epithelial and mesenchymal cells, following long-term DR [49]. However, while frequencies of senescent cells increased during aging in skin of rhesus monkeys [50] and baboons [25], no decrease of sen-β-Gal-positive epithelial cells and no increase in proliferation-competent skin fibroblasts was found after 9-12 years of DR in rhesus monkeys [50]. To our knowledge, there is no data reporting an effect of shorter term DR on cell senescence in solid tissues.

We tested the impact of short-term (3 months), adult-onset DR on cellular senescence in mice. We concentrated on the small intestine, a highly proliferative organ, and on liver with a slow cell turnover under non-pathological conditions. We had shown before that senescent cell frequencies in these organs increase significantly during normal aging in mice [28]. Using sensitive and specific markers for senescent cells [51,52], we found that short-term, adult-onset DR significantly reduced the frequencies of senescent liver hepatocytes, especially in the centrilobular area, and of senescent intestinal enterocytes in the transient amplifying zone. DR also improved telomere maintenance in liver and intestine and reduced cumulative oxidative stress markers in the same tissue compartments. We propose that reduction of cell senescence might be a primary effect of DR which may explain improved mitochondrial function and reduced ROS production.

## RESULTS

### Adult-onset, short-term DR reduced the frequencies of senescent cells in small intestine and liver

Male C57/BL mice were subjected to three months of DR by average 26% of food restriction starting at 14 months of age. The study cohort is characterized in supplementary Table S1. We focused on intestinal crypt enterocytes and liver hepatocytes because frequencies of senescent cells in these tissue compartments increased with age or as result of telomere dysfunction in Terc-/- mice [23,28].

**Figure 1.**
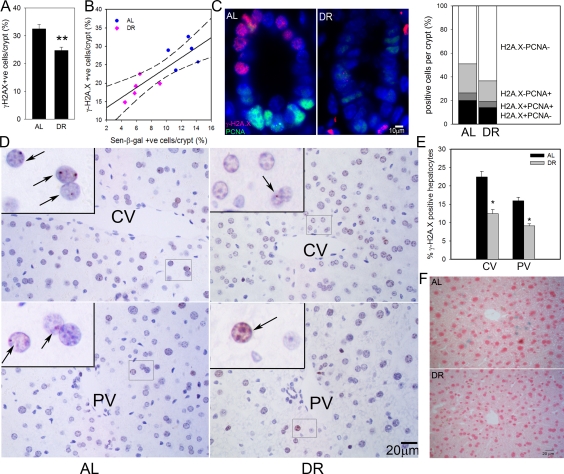
DR reduced frequencies of senescent hepatocytes and intestinal crypt enterocytes. (**A**) Frequencies of γ-H2A.X positive enterocytes per crypt, immunohistochemistry on paraffin sections. ** p<0.005. (**B**) Correlation between sen-β-Gal and γ-H2A.X positive enterocytes (p=0.002). Data points are means per animal (DR: pink; Al: blue). Linear regression (solid line) and 95% confidence intervals (dashed lines) are given. (**C**) Representative images (left) and quantitative evaluation (right) of PCNA and γ-H2A.X double immunofluorescence of intestinal crypts from AL and DR mice. Blue: DAPI; red: γ-H2A.X; green: PCNA. (**D**) Representative images of γ-H2A.X immunohistochemistry in livers from AL (left) and DR (right) mice. Examples of centrilobular (top) and periportal (bottom) areas are shown. CV: central vein; PV: portal vein. Boxed areas are shown at higher magnification. Arrows indicate nuclei containing γ-H2A.X foci (red). (**E**) Quantification of γ-H2A.X positive hepatocytes. * p<0.05. (**F**) Representative images for sen-β-Gal activity. Pink: nuclei; blue: cytoplasmic sen-?-Gal staining. All data are from 5 animals/group, mean±S.E.M.

We first measured the frequency of intestinal enterocytes showing an active DNA damage response as characterized by nuclear positivity for the DNA damage marker, γ-H2A.X. As we have shown before, there were few γ-H2A.X-positive enterocytes within villi, instead, positive cells centered around the transient amplifying zone in crypts [28]. DR significantly reduced the frequencies of γ-H2A.X-positive intestinal crypt enterocytes (Figure 1A). We compared frequencies of γ-H2A.X-positive and sen-β-Gal-positive crypt enterocytes, measured on adjacent frozen sections from five AL and five DR mice (Figure 1B). The significant reduction of positive cells by DR was confirmed for both markers, and they were significantly correlated (r^2^=0.7080). γ-H2A.X staining on its own may overestimate frequencies of senescent cells, especially in tissue compartments with high proliferative activity such as gut because an active DNA damage response can also be initiated by replication stress in dividing cells. Accordingly, we showed recently that a combination of strong positivity for γ-H2A.X with absence of a proliferation marker results in quantitatively correct estimates of senescent cell frequencies *in vitro* and *in vivo*[52]. Double staining for γ-H2A.X and PCNA in the small intestine (Figure 1C) showed that DR reduced also the frequencies of PCNA positive crypt enterocytes as reported previously [45]. Frequencies of γ-H2A.X positive/PCNA negative intestinal crypt enterocytes in 17 month old mice were 20.0±0.9% under AL conditions and 14.0±1.9% after 3 months DR (Figure 1C). This difference was significant (p=0.02).

In liver, frequencies of γ-H2A.X positive hepatocytes were higher in centrilobular than periportal areas (Figure 1D) as shown previously [28]. Importantly, the frequencies of γ-H2A.X positive hepatocytes were significantly reduced following 3 months DR by 6.5 ± 1.8% in the centrilobular area and by 3.3 ± 1.2% in the periportal area (Figure 1E). Results were qualitatively confirmed by sen-β-Gal staining on cryosections (Figure 1F). The frequency of PCNA- or Ki67-positive cells in hepatocytes was less than 1% (data not shown). Therefore, γ-H2A.X positivity on its own is regarded as a good estimate of senescent hepatocytes in liver.

### Adult-onset, short-term DR improved telomere maintenance in small intestine and liver

**Figure 2.**
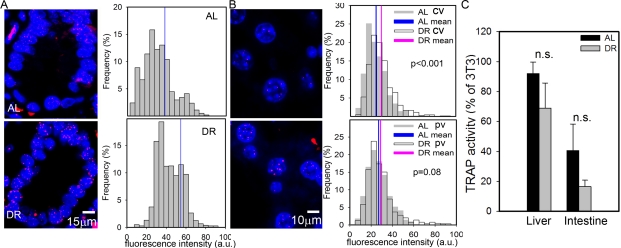
DR improves telomere maintenance. (**A**) Representative Q-FISH images (left panels, red: telomeres, blue: nuclei) and distribution of enterocyte telomere fluorescence intensity per nucleus (right panels, n≥2230 nuclei, 5 animals) in intestinal crypts. Mean nuclear telomere fluorescence intensity is indicated by blue vertical lines. p<0.001, Mann-Whitney rank sum test. (**B**) Representative Q-FISH images (left panels, red: telomeres, blue: nuclei) and distribution of hepatocyte telomere fluorescence intensity in centrilobular (CV, top, n≥560 nuclei) and periportal (PV, bottom, n≥650 nuclei) in liver areas. Mean fluorescence intensities are indicated for AL (blue) and DR (pink). P-values for AL vs DR were calculated by Mann-Whitney rank sum test. (**C**) Telomerase catalytic activity (% of TRAP activity in 3T3 cells) in whole liver (left, n=4) and intestinal mucosa (right, n=5) homogenates. Data are mean±S.E.M. n.s.: not significant (T-test).

Despite the presence of active telomerase, telomeres shorten with age in various tissues of laboratory mice [28,53]. However, even in very old mice, telomeres are much longer than in humans and aging in mice did not measurably increase the degree of co-localisation of DNA damage foci with telomeres [28]. This suggests that telomere shortening may only be a minor contributor to cell senescence in aging wild-type mice. Here, we measured telomere length by quantitative FISH (Q-FISH) in intestinal enterocytes and liver hepatocytes (Figure 2A, B). Following 3 months of DR, the average telomere length per crypt enterocyte nucleus was significantly higher than in AL fed mice (Figure 2A). The effect of DR on hepatocyte telomere length was smaller than in the intestine (Figure 2B), possibly because of the lower rate of proliferation. However, the difference between DR and AL was still significant in the centrilobular areas. Telomerase activity as measured by TRAP in whole liver and intestinal mucosa homogenates was not significantly changed by DR (Figure 2C). If anything, it tended to decrease under DR, possibly due to the anti-proliferative effect of DR, suggesting that other factors than telomerase must be responsible for the improved telomere maintenance under DR. The most probable of these is reduction of oxidative damage to telomeres [54].

### Adult-onset, short-term DR reduced some oxidative damage markers in small intestine and liver

Senescent cells are a major source of ROS because mitochondrial dysfunction and, possibly, other ROS-producing mechanisms are part of the senescent phenotype [23,55-57]. Long-term DR is well known to reduce oxidative stress and mitochondrial ROS production [8,58]. We measured several markers of oxidative damage in small intestine and liver to test whether adult-onset, short-term DR impacts on oxidative stress in the same tissues as it reduced cell senescence.

**Figure 3.**
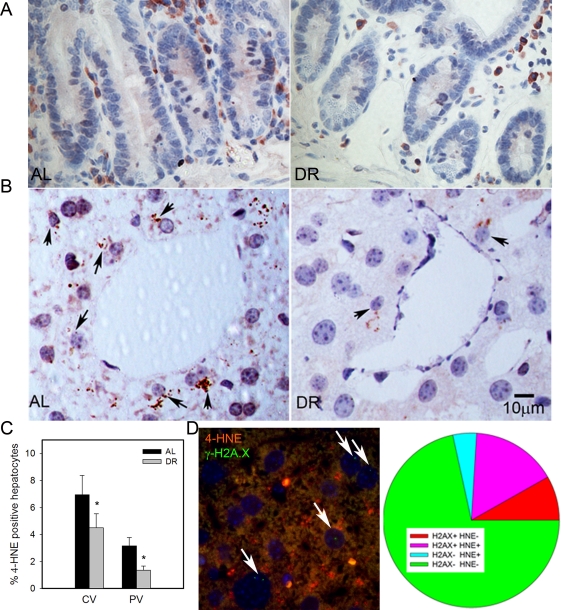
DR decreased lipid peroxidation in liver. (**A**) Representative 4-HNE immunohistochemistry in small intestine from AL (left) and DR(right) mice. Brown: 4-HNE staining; blue: nuclei. (**B**) Representative 4-HNE images from centrilobular areas in liver. Brown: 4-HNE, Blue: nuclei. Arrows indicate examples of positive cells. (**C**) Frequencies of 4-HNE-positive hepatocytes in periportal and centrilobular areas of liver. Data are mean±S.E.M. * p<0.05, n=5 animals/group. (**D**) Co-localisation of γ-H2A.X (green) and 4-HNE (red) in AL liver. Representative image, double immunofluorescence, cryosection. Cells with nuclei (DAPI, blue) positive for γ-H2A.X are marked by arrows. Cells were scored as either single positive (H2AX+ HNE - or H2A.X- HNE +), double positive (H2A.X+ HNE+) or double negative (H2A.X- HNE -). Data are from four animals from the AL group.

4-HNE is a major end product of lipid peroxidation and has been shown to accumulate in tissues with age [59]. We found few 4-HNE positive cells in intestinal crypts, and almost all were located in the lamina propria (Figure 3A). Confirming earlier results [28], HNE-positive hepatocytes were more frequent in centrilobular than in periportal areas. Importantly, frequencies of HNE-positive hepatocytes decreased under DR in both areas (Figure 3B, C p<0.05). To directly see whether there was an association between cell senescence and oxidative stress in liver hepatocytes, we performed a double staining for γ-H2A.X and 4-HNE (Figure 3D). Quantitative evaluation showed that the majority of senescent hepatocytes (as measured by γ-H2A.X) were also positive for 4-HNE and, vice versa, about three quarters of 4-HNE-positive hepatocytes were probably senescent (Figure 3D), thus confirming a cell-specific association between senescence and a marker of oxidative damage.

**Figure 4.**
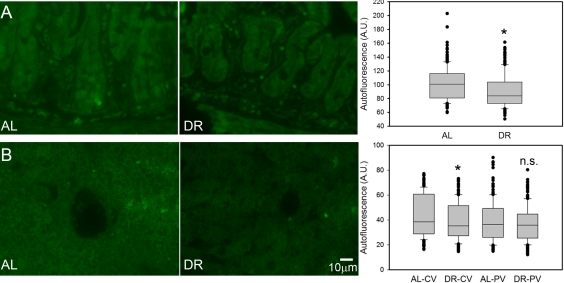
DR decreased the intensity of broad-band autofluorescence. (**A**) Representative autofluorescence images (left) and quantitative data (right) in small intestinal crypts under AL (left) and DR (right). (**B**) Representative autofluorescence images from centrilobular areas in liver (AL left, DR right) and quantitative data in periportal and centrilobular areas. All data are mean±S.E.M from 5 animals/group. *p<0.05; n.s. not significant (T-test).

Broad-band autofluorescence originates mainly from oxidised and cross-linked cell components, like advanced glycation end products (AGEs) and lipofuscin and is thus regarded as a good cumulative marker for oxidative damage [23, 60-62]. Short-term DR significantly reduced the intensity of broad-band autofluorescence from intestinal crypt enterocytes (Figure 4A) and in centrilobular areas of the liver (Figure 4B). The reduction of autofluorescence in the periportal areas of the liver by DR was not significant (Figure 4B), in accordance with this compartment showing the least reduction of senescent cells.

**Figure 5.**
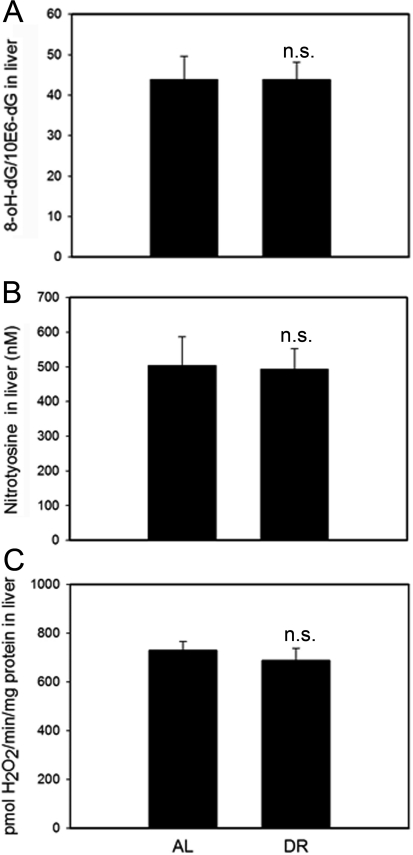
DR does not change oxidative damage markers measured in whole liver homogenates. (**A**) 8-oxodG levels in liver homogenates from AL and DR mice measured by HPLC with electrochemical detection. n=9 animals/group. (**B**) Nitrotyrosine levels in liver homogenates from AL and DR mice measured by ELISA; n=6 animals/ group. (**C**) Steady state hydrogen peroxide release from liver homogenates from AL and DR mice measured by Amplex Red fluorimetry; n=12 animals/ group. All data are mean ±S.E.M.; n.s.: not significant (T-test).

8-oxodG (a marker for oxidative DNA damage), nitrotyrosine content (a marker for oxidative protein damage) and H_2_O_2_ release rate from tissue homogenate are indicative of steady-state levels of oxidative stress/oxidative damage. These markers were measured in whole liver homogenates. None of them were significantly different between AL and DR mice (Figure 5). Similarly, DR did not change 8-oxodG levels in homogenates of the intestinal mucosa (data not shown).

## DISCUSSION

This is the first study to show that short-term DR reduced frequencies of senescent cells in solid tissues. It is important to note that the magnitude of the reductions, amounting to between 3.3 and 6.5% depending on the tissue compartment, is very substantial given the short duration of the treatment. Frequencies of senescent cells increase with age in intestinal crypts and liver at rates below 0.5% per month [28], indicating that 3 months DR probably reduced levels of senescent cells beyond that at the start of the treatment. Available data indicate that senescent hepatocytes are turned over slowly in liver [63,64]. Turnover rates of senescent enterocytes in intestinal crypts are unknown. DR could block the induction of senescent cells, increase the rate of their turnover, or both.

Cell senescence *in vitro* is associated with a 3- to 5-fold increase in cellular ROS levels [23,55-57]. Various signaling pathways and feedback loops connect DNA damage response and checkpoint proteins that are activated early and permanently in senescence, notably p21, p16 and Rb, with ROS generation via mitochondrial dysfunction and, potentially, NADPHoxidase activation [23,56,65]. As senescent cells were less in DR, we therefore expected to see lower levels of oxidative stress markers under DR especially in those tissue compartments which showed large reductions of senescent cells. This was indeed the case: auto-fluorescence was significantly reduced in the intestinal crypts and the centrilobular areas of the liver, but not around the portal vein. While 4-HNE could not be measured in enterocytes, it was more strongly reduced around the central vein than in the periportal areas of the liver. Without increases in telomerase activity, telomere length was better maintained under DR in the crypt enterocytes and in the centrilobular, but not periportal, areas of the liver. Autofluorescence, 4-HNE and telomere maintenance in the absence of changes in telomerase activity are all regarded as cumulative markers of oxidative damage [54,59, 62]. In contrast, we did not find any significant effect of DR on the markers of oxidative damage measured in whole tissue homogenates. This was not surprising because 8-oxodG and H_2_O_2_ release as acute parameters are less sensitive than the cumulative markers mentioned above. Moreover, an average decrease in the number of senescent cells by about 5% in liver would result in less than 10% decrease in total ROS, which is within the experimental error for these measurements. As there are very few senescent cells in villi, the expected impact of the observed decreases in senescent cell frequencies in the crypts on ROS in the whole intestinal mucosa would be even lower.

Taken together, our data suggest that at least some of the beneficial effects of DR that have been repeatedly described in the literature, such as improved mitochondrial coupling and reduced ROS release [8,58], could be quantitatively explained as an indirect effect, mediated via reduction of senescent cells. Mitochondria are dysfunctional (i.e. produce more superoxide despite lower membrane potential and induce a retrograde response) not only in senescent human fibroblasts *in vitro*[57]. The same changes were triggered by telomere dysfunction in mouse cells and tissues including the intestinal crypt epithelium. In this system, as in human fibroblasts, mitochondrial dysfunction was dependent on signaling through p21, the central mediator of cell senescence [23,32].

Our results lead to the question of how DR could impact directly on cell senescence. One interesting candidate may be signaling through the mTOR-S6K1 pathway. DR reduced phosphorylation and activity of Akt1, mTOR and its downstream targets S6K1 and 4E-BP1 [66]. Knockout of S6K1 mimics the effects of DR [67]. Importantly, S6K1 is intimately involved in the regulation of cell senescence. S6K1 phosphorylation and activity is altered in replicative senescence [68]. mTOR activation induced senescence in human fibroblasts [69] and the activation state of the mTOR pathway has been shown to be relevant for the decision between reversible arrest and cell senescence in models of DNA damage-independent senescence [70]. Wnt1-driven activation of the mTOR pathway caused epithelial stem cell senescence and loss after a short hyperproliferative period [71]. A mechanistic clue comes from a recent paper showing that activated S6K1 binds more tightly to Mdm2, inhibiting Mdm2-mediated p53 ubiquitination and thus stabilizing p53-dependent DNA damage signaling [72]. Accordingly, suppression of mTOR-S6K1 signaling as occurring under DR would lead to Mdm2 nuclear transduction, activate p53 degradation and reduce thus signaling towards apoptosis and/or senescence.

In conclusion, the data are compatible with the idea that reduction of cellular senescence is a primary effect of DR, possibly mediated via suppression of signaling through mTOR-S6K1, and that this reduction in turn might be sufficient to account for the improvement of mitochondrial function and reduction of ROS production that are known to occur under DR.

## METHODS

### Animals

From a group of 90 male C57/BL mice aged 14.2 ± 1.2 months, 45 animals were subjected to DR, while the other 45 animals, matched for body mass, food intake and age, served as ad libitum-fed (AL) controls. The experiment lasted for 3 months with an average food restriction of 26%. All mice were sacrificed at the end of the experiment. Five mice per group were perfused by whole animal fixation with 4% paraformaldeyde followed by dissection. Tissues were paraffin-embedded and 5μm sections were prepared from small intestine and liver. Tissues from five additional mice per group were frozen in OTC for cryosectioning. Tissues from further animals were frozen in liquid N_2_. The intestinal mucosa was stripped from the muscle layer before freezing. Further details of the experimental protocol can be found as supplementary material, Table S1. The project was approved by the Faculty of Medical Sciences Ethical Review Committee, Newcastle University.

### Histochemistry, Immunofluorescence, Telomere Q-FISH and telomerase activity

Sen-β-Gal histo-chemistry, immunohistochemistry and telomere Q-FISH were performed as described [28]. The antibodies used and the dilution factors were: anti-γ-H2A.X (#9718, Cell Signalling, Herts, UK, 1:250), anti-PCNA (#ab27Abcam, Cambridge, UK, 1:1,000) and anti-4-HNE (#MHH-030n, Japan Institute for the Control of Aging, Japan, 1:500). Incubation with all primary antibodies was overnight at 4°C.

For double immunofluorescence, blocked sections were incubated with anti-PCNA and anti- γ-H2A.X anti-bodies together in PBS at 4°C overnight and incubated with Alexa-555-conjugated goat anti-rabbit antibody and biotinylated anti-mouse antibody for 45 min in PBS. Subsequently, tissue sections were washed 3 times and incubated with 0.2% Fluorescein Avidin-DCS in PBS for 30min. Images were taken in a Leica DM5500B microscope with 40x objective. 30-40 crypts were scored for each animal.

Telomerase activity was measured using the TeloTAGGG Telomerase PCR ELISA kit (Roche) according to the manufacturer's recommendations.

### Autofluorescence

Autofluorescence was measured on unstained, non-deparaffinized tissue sections using a Leica DM5500B microscope. The sample was excited at 458nm and fluorescence emission captured above 475nm.

### 8-oxodG

The base oxidation product 8-oxo-7,8-dihydro-2'-deoxyguanosine (8-oxodG) was detected by HPLC with electrochemical detection (ECD). Ground frozen tissues (30-100 mg, n=4-9 per group) were thawed and genomic DNA was obtained using standard phenol extraction [73]. The DNA extraction procedure was optimized to minimize artificial induction of 8- oxodG, by using radical-free phenol, minimizing exposure to oxygen and by addition of 1 mM deferoxamine mesylate and 20 mM TEMPO (2,2,6,6-tetramethylpiperidine-N-oxyl), according to the European Standards Committee on Oxidative DNA Damage [74]. HPLC-ECD was based on a method described earlier [75]. Briefly, 30 μg DNA was digested to deoxyribonucleosides by treatment with nuclease P1 [0.02 U/μl] for 90min at 37 °C and subsequently with alkaline phosphatase [0.014 U/μl]. for 45min at 37 °C. The digest was then injected into a Gynkotek 480 isocratic pump (Gynkotek, Bremen, Germany) coupled with a Midas injector (Spark Holland, Hendrik Ido Ambacht, the Netherlands) and connected to an Supelcosil^TM^ LC-18S column (250 X 4.6 mm) (Supelco Park, Bellefonte, PA) and an electrochemical detector (Antec, Leiden, the Netherlands). The mobile phase consisted of 10% aqueous methanol containing 94 mM KH_2_PO_4_, 13 mMK_2_HPO_4_, 26 mM NaCl and 0.5 mM EDTA. Elution was performed at a flow rate of 1.0 ml/min with a lower absolute detection limit of 40 fmol for 8-oxo-dG, or 1.5 residues/10^6^ 2'-deoxyguanosine (dG). dG was simultaneously monitored at 260 nm.

### Nitrotyrosine measurement

Ground frozen tissues (8-32mg, n=5 per group) were thawed and total protein was extracted using Microplate BCA^TM^ protein assay kit (Thermo Scientific, UK). Nitrotyrosine was detected by oxiSelect^TM^ Nitrotyrosine ELISA kit (Cell Biolabs, INC, UK) according to the protocol provided by the manufacturer.

### H_2_O_2_ release

Ground frozen tissue was homogenized in PBS and used immediately for the assay. The rate of hydrogen peroxide release was monitored fluoro-metrically as resorufin formation due to oxidation of Amplex Red (10-acetyl-3,7-dihydroxyphenoxazine, purchased from Invitrogen, 50μM) in the presence of horseradish peroxidase (2U/ml), at an excitation 544 nm and an emission 590 nm using a FLUOstar Omega (BMG Labtech). Superoxide dismutase (75U/ml) was included in the assay buffer. The slope was converted into the rate of hydrogen peroxide release with a standard curve. Protein concentration was measured using Bio-Rad DC protein assay kit.

## SUPPLEMENTAL MATERIAL

Table S1. Characterisation of the experimental cohort
