# Supplementary material for: Adult-onset, short-term dietary restriction reduces cell senescence in mice
Source: Aging (Albany NY). 2010 Sep 11;2(9):555–66. doi: 10.18632/aging.100196 (PMC2984605; doi:10.18632/aging.100196)
Supplement: Table S1. [file aging-02-555-s001.doc]

Parameter	Ad libitum (AL)	Dietary restriction (DR)	P - value	
Mean age at death (months)	17.68±1.21	16.74±0.97	<0.001	
Animal deaths during experiment other than tumours	3	2	0.738	
Macroscopic tumour incidence	6	2	0.150	
Body mass (g) 	39.38±0.37	34.26±2.24	<0.001	
Food intake (g) 	3.59±0.41	2.67±0.00 	<0.001	
Mean daily body temperature (oC) 	35.98±0.11	35.72±0.09 	0.002	
Mean daily physical activity (arbitrary units) 	13.64±2.33	19.63±13.44 	0.252	
